# Rethinking the relationship between science and society: Has there been a shift in attitudes to Patient and Public Involvement and Public Engagement in Science in the United Kingdom?

**DOI:** 10.1111/hex.12295

**Published:** 2014-10-31

**Authors:** Annette Boaz, Despina Biri, Christopher McKevitt

**Affiliations:** ^1^ Faculty of Health, Social Care and EducationKingston University and St George's, University of LondonLondonUK; ^2^ Department of Science, Technology, Engineering and Public Policy (UCL STEaPP)University College LondonLondonUK; ^3^ King's College LondonLondonUK

**Keywords:** attitudes, biomedical research, Mode 1 and Mode 2, Patient and Public involvement, Public Engagement in Science, scientists

## Abstract

**Background:**

The policy imperative to engage the public and patients in research can be seen as part of a wider shift in the research environment. This study addresses the question: Has there been a shift in attitudes to Patient and Public Involvement (PPI) and Public Engagement in Science (PES) amongst researchers?

**Methods:**

Attitudes to PPI and PES within a cluster of three NIHR supported Biomedical Research Centres were explored through in‐depth interviews with 19 researchers.

**Results:**

Participants distinguished PPI (as an activity involving patients and carers in research projects and programmes) from PES (as an activity that aims to communicate research findings to the public, engage the public with broader issues of science policy or promote a greater understanding of the role of science in society). While participants demonstrated a range of attitudes to these practices, they shared a resistance to sharing power and control of the research process with the public and patients.

**Conclusion:**

While researchers were prepared to engage with the public and patients and listed the advantages of engagement, the study revealed few differences in their underlying attitudes towards the role of society in science (and science in society) to those reported in previous studies. To the participants science remains the preserve of scientists, with patients and the public invited to ‘tinker at the edges’.

## Background

Traditionally, the concepts of Patient and Public Involvement (PPI) in research and Public Engagement in Science (PES) have occupied parallel tracks. PES has a long history as scientists have sought to educate the public about science in general and their own scientific research in particular; restore public trust in their activities, and obtain and maintain a ‘license to practice’.^1^ PPI in health research, as formally constituted in the UK and other mainly English speaking countries, is a newer phenomenon seeking variously to promote participatory democracy; increase accountability of scientific researchers; and recognize and draw on experiential knowledge and expertise of lay users of health care.^2^ Both might be seen as part of a broader trend towards reconceptualizing the role of society (i.e. citizens, patients and the public) in science. Nowotny and colleagues^3^ point to the introduction of systems of accountability, the steering of research priorities (for example through the EU Framework programmes) and the emphasis on commercialization as signs of a changing research environment and a shifting role for science and scientists.

Some signs of a shift are evident in the academic literature. For example, Wynne^4^ explicitly calls on scientists and scientific institutions to democratize the production of knowledge. Engagement as a strategy for accountability is also characterized as a response to a supposed crisis in trust leading to a so‐called democratic deficit in scientific research.^5^ For Weldon,^6^ a more accountable science will reverse the decline of trust in scientists and scientific institutions. Nilsen^7^ cites the WHO Alma Ata declaration in support of claims that ‘participation’ in planning, organization, operation and control of health care, encourages democracy, accountability and transparency. Boote *et al*.^8^ argue that as UK citizens are financial contributors to and therefore part owners of the NHS they have a right to have a voice in NHS activities and processes, including research.

Alongside this academic debate, a set of policy initiatives have encouraged greater PPI in health research.^9^ There has also been a significant investment in PES (particularly supported by science funders such as the UK Wellcome Trust) and activity by scientists to respond to public concerns about scientific issues. While PES is a more established concept for biomedical researchers, PPI has a growing currency particularly given its profile with funding bodies such as the UK National Institute for Health Research (NIHR). Furthermore, the promotion of so‐called translational research, with its focus on investing in research that will yield applications in clinical practice also marks a shift towards what Gibbons describes as ‘Mode 2’ research.^10^ While Mode 1 is characterized by the autonomy of scientists and the dominant role of scientific discovery, Mode 2 is described as multidisciplinary, socially distributed and orientated towards application and use. With this shift comes a renewed emphasis on both engagement and application.^3^

There have been few studies exploring the responses of researchers to these shifts. Those studies that have focused on researcher responses^11^, ^12^, ^13^, ^14^ have described researchers seeking to accommodate policy requirements such as stakeholder engagement and promoting implementation, but experiencing a ‘pull back’ to Mode 1 from their disciplines and institutions.^11^ Previous studies have observed the defensive power of the research community.^11^, ^12^ Ferlie and Wood^11^ describe the separation of research and implementation in one clinical research team, allowing for high quality research publications to continue to take priority. Ward *et al*. identified an epistemological dissonance in which researchers continue to privilege expertise over experience, attributing little value to lay knowledge.^14^ In the Public Understanding of Science literature, social scientists also discern changes in scientific activities and behaviour while ‘the deeper tidal rhythms of science and its governance remain resistant’.^15^ Given the recent policy shifts relating to PPI and efforts to promote greater engagement of citizens in the production of scientific knowledge, this paper investigates whether there has been a blurring in the distinction between science and society. In particular, this study provides an opportunity to explore the attitudes of academic researchers to the changing research environment and the shifting roles of science and scientists, focusing on researchers located in UK NIHR funded Biomedical Research Centres (BRCs). We look in particular at their responses to initiatives designed to increase engagement with the public and patients and ask: is the shift in policy and practice around PPI and PES reflected in a shift in attitudes amongst researchers?

## Methods

Biomedical Research Centres offer an interesting research setting for a study of PPI and PES: they are designed to conduct ‘bench to bedside’ translational research, speeding up the process of bringing new treatments for patients from the laboratory to the clinic^16^ and they include a range of types of research and researcher. Furthermore, the NIHR funding for these flagship initiatives is accompanied by requirements to adhere to NIHR guidance on PPI. As part of one BRC, two of the authors (CM and AB) had been tasked with conducting research on PPI practice in the BRC and had observed that basic and some clinician scientists often conflated PPI and PES. Given this observation, this study explored researchers' attitudes to both PPI and PES within a cluster of three NIHR supported BRCs, through a series of in‐depth interviews.

Participants were identified from staff lists provided by the administrative teams in the three BRCs. Participants were selected from one research theme within each centre with the aim of including both different types of researchers and staff at different levels, ranging from relatively junior staff (research associates and PhD students) through to professors. Nineteen researchers were drawn from three research themes: genetics, mental health and health services research. The sample included a mix of basic biomedical scientists, health service researchers and clinician scientists (engaged in both research and clinical practice). Details of the sample are included in Table 1. Semi‐structured interviews were conducted, using a topic guide covering the following broad themes: experiences and perceptions of PPI and PES and benefits and challenges to PPI and PES. To assess understandings of PPI and PES practices, the participants were also asked to carry out a card sorting exercise, in which they were asked to categorize a set of cards listing PPI and PES activities most commonly given in the literature (as determined through an extensive review of the PPI and PES literatures) into those they considered to be typical of PPI and those they considered to be typical of PES. They were encouraged to share the reasoning behind their choice during the exercise, and were free to redesignate activities to the other category if they so wished, or to not assign a category if they felt the activity was not typical of either PPI or PES. In addition, a card listing commonly given reasons for PPI and PES derived from the literature was also used to stimulate discussion. All interviews were conducted by DB. Interview transcripts were entered into NVivo9. The initial coding was generated inductively from close reading of the transcripts and then grouped into broad themes that were augmented in the analysis to take account of emerging interpretations. Gibbons' type 1 and type 2 theory was used at this stage to further interrogate the data.^10^ Analysis involved a process of constant comparison, with particular emphasis given to deviant cases, with the aim of developing interpretation and explanation. This study was approved by the Biomedical & Health Sciences, Dentistry, Medicine and Natural & Mathematical Sciences Research Ethics Committee at King's College London.

**Table 1:** Interview participants

Pseudonym	Post	Research area
Janet	Professor	Health Services Researcher – Innovations
Allan	Professor	Biomedical Scientist (Clinician Scientist) – Mental Health
Roger	Professor	Biomedical Scientist – Genetics
Trevor	Professor	Biomedical Scientist – Mental Health
Peter	Reader	Biomedical Scientist – Genetics
Raj	Senior Lecturer	Health Service Researcher (Clinician Scientist) – Mental Health
Sue	Research Fellow	Health Service Researcher – Innovations
Anna	Research Fellow	Health Service Researcher – Innovations
Sam	Research Associate	Health Service Researcher – Innovations
Philip	Research Associate	Health Service Researcher – Innovations
Jackie	Research Associate/ PhD student	Health Service Researcher – Innovations
Ewan	Post‐doctoral Researcher	Biomedical Scientist – Genetics
Tanya	Post‐doctoral Researcher	Biomedical Scientist – Genetics
James	Post‐doctoral Researcher	Biomedical Scientist – Genetics
Jack	Post‐doctoral Researcher	Health Service Researcher – Mental Health
Zoe	PhD student	Biomedical Scientist – Genetics
Tom	PhD student	Health Service Researcher – Mental Health
Jude	PhD student	Health Service Researcher – Mental Health
Shaun	Research Assistant	Health Service Researcher – Mental Health

## Findings

While participants had varying levels of experience of PPI and PES, they were generally clear on the definitions of each. When completing the card sorting exercise, they were consistent in their categorizations of PPI and PES methods and activities. Participants made a distinction between PES and PPI in terms of the level at which they were undertaken, their purpose and those who were involved. Broadly speaking, they considered PES to be concerned with communications with the broader public, often about science more generally (through, for example giving talks in schools about the value and contribution of science to society) and improving public understanding of science through for example media appearances. PPI was considered to relate to involvement in individual research projects and programmes. Participants described a range of activities including consulting patient groups on ethics applications, inviting patients to join advisory groups, and involving patients as researchers. Despite the label PPI, these activities were typically associated with patients and carers and not with the wider public.

You are talking about patients and public and I think the two things need to be separated (Sue)

The differences between participants lay in their underlying attitudes. The attitudes to PPI expressed in the interviews fell into two main categories: those who were positive about some involvement and those who were pragmatically accommodating PPI. Despite the different positions of the two groups, they shared a resistance to sharing power and control in the process of knowledge generation. The attitudes to PES were relatively consistent across the interviews. Participants were concerned with promoting a better understanding and acceptance of the role of science in society. A single participant expressed a much more supportive view of the role of patients and the public. The following sections discuss these four sets of attitudes in turn.

### Positive attitudes to PPI: ‘You get better research’

All participants were able to identify a wide range of potential outcomes for activities designed to involve patients and the public including promoting public awareness of research, improving research recruitment and increasing the relevance of research. However, some participants talked more positively of the importance of PPI and this group perceived of some benefits to engaging with patients and the public. In particular, health services researchers referred to the potential to improve the quality of research tools, questions, processes and outcomes.

I think it's really, really, really important, and the research I think that's important, because you get better research, you get better questions, you get challenged, you design your studies differently, you choose different outcome measures…' (Janet)

Another participant supported this view, describing the potential for the views of patients and the public to be ‘like dynamite’ in changing thinking and practice. Those who identified benefits often spoke of their own personal positive experiences. For example, one professor experienced in PES commented on his more recent PPI activities:

[We] have become very aware of the need to involve users and participants and we've sought their advice and help to design better studies, to help with the recruitment, to help understand the experience of the subject of research, so we've been involving them all the way along. Prior to that, I didn't give it sufficient attention in my research, so it's something I've, sort of, become more aware of in recent years. I mean, in terms of public engagement, I have been involved with some research that's had quite a high profile and I have been involved with press releases and writing articles for the sort of lay press as well as the sort of scientific press. So that's been of interest and I think I kind of understand how to communicate with the general public. Well, I've been better since having had to do it. (Trevor)

Some participants referred to the positive impact of being exposed to new perspectives and patient experiences. Janet felt that involving patients in her research had led to better research questions, the selection of more appropriate designs and outcome measures and had brought an element of challenge to the research process. Sam described how involving parents in a study of support for physically disabled children in mainstream schools made a difference in terms of implementation as the parents were keen to avoid the findings gathering dust in a library. This motivated the researchers to secure additional funding to produce a booklet for schools based on the findings.

Yet, they also shared a number of challenges based on their experience, including difficulties recruiting service users to be ‘involved’, and the need for research training for patients and the public. Two participants commented on the significant amount of work that had to be performed before the service users could get more actively involved in research (including recruitment, obtaining honorary contracts etc.). In one case, recruitment to the study had finished by the time the service user was in post. Recruitment was the aspect of the project where the researcher had planned for the service user to contribute and for which she had obtained ethics approval. The researcher needed to think again about how to include the service user, how the service user might like to be involved and whether this change would have implications for ethics.

I was doing a project where we were trying to get service users on board, and we had employed two, from the [local patient group], so we had gotten them on board, got honorary contracts for them through [the Trust], and then, of course, recruitment stopped. So I was kind of left at a loose end of what to do with them. (Shaun)

In this case, the researcher was left searching for an ‘appropriate’ task within the project for the service user researchers.

### Pragmatic accommodation of PPI: ‘You just need the box ticked’

Many interview participants expressed disinterest in or active hostility to PPI. Typically, they described PPI activities as things that ‘had to be done’. Raj, a senior lecturer described the funding imperative to demonstrate involvement.

it's more or less for any funding I think, nowadays you have to demonstrate some sort of engagement (Raj)

PPI was described as a bureaucratic activity designed to fulfil policy and funding requirements. One participant in this group went as far as to describe PPI as a scripted activity, in which patients and the public are encouraged to play a predetermined, bounded role in the research process:

With public involvement, it always seems to me that you're promising people far more than you're ever going to deliver. That you're encouraging them to feel engaged because that's the role that you scripted for them and you want them to play that part. But really once the study ends or that part of it ends you don't really want much more to do with them. (Sam)

Participants expressed concern about the skills of patients and the public and their ability to engage meaningfully in research. One participant highlighted the difficulty in involving the public in molecular biology at the bench end of the translational pipeline, compared to clinical trials where the relevance to patients is more obvious and there may be scope for more patient involvement. A health service researcher also referred to the translational pipeline, expressing the view that health service research (located closer to the bedside) provides more opportunities to take on board and value patient perspectives.

Furthermore, biomedical scientists stressed the technical nature of their particular areas of science and argued that specialist knowledge was required to play an active role in research.

But how could somebody who's not a scientist have influence in the design of any sort of experiment? Because… you know what I mean? It's hard enough for us to design an experiment so it actually works and it makes sense, it's hard work, and you need to understand what you're doing. And, I cannot imagine that the general public would have any sort of positive impact on that. (Euan)

The biomedical scientists in particular were also anxious about relinquishing any control over the research process to non‐scientists:

I guess, I'm also a bit scared of this idea of handing over some of the power and control to the public so they can influence how research is conducted, because, I feel like the decisions would be quite naive. It may not necessarily be in the best interests of research progress or, you know, getting a new drug or something like that. (Tanya)

I respect other people's opinions and they need to respect mine. In terms of whether the experiments are the right ones to do, then I think that's something you have to do within your peer group. I think that's not really… you're not going to get a better experiment design by talking to someone who doesn't understand how to do an experiment. (Peter)

The threat of losing control of the scientific process to patients and the public loomed large in the interviews with the laboratory based biomedical scientists. For these participants there was no ‘blurring’ of the distinction between science and society. With a couple of exceptions, health service researchers also became uncomfortable when confronted with the idea of sharing power and control with patients and the public, highlighting the danger of ‘devaluing research skills’, ‘mixing up roles’ and ‘representation’.

So I think for me, it's absolutely fine for patients to have enormous power over the direction of research and what the questions are, but the technical sides of it are I don't think appropriate for patients. And then you get confused about the sorts of patients you're attracting, and what actually is a lay person if they're someone that's capable of carrying out a scientific study…So I think it's just a mixing up of roles that isn't terribly helpful to anyone. (Sam)

### Attitudes to PES: ‘Getting people to understand better what we do.’

Consistent with their attitudes to PPI, the biomedical scientists and some health services researchers associated PES with a more traditional notion of public understanding of the role of science in society. They talked about their experiences of going into schools to encourage young people to pursue careers in science and attending science fairs to promote public understanding of and trust in science. They also discussed their experiences of science communication, and in particular the difficult relationship they perceived between science and the mass media.

I guess it's the idea of ensuring that people understand what it is that scientists do, and what our research means, so that we're not just…so that academia doesn't become something that's self‐serving, or a game, but that people outside of the academic world understand what that means. (Jude)

This conceptualization of PES was consistent across the different levels of experience within the genetics and mental health themes. Some participants felt scientists could do more in terms of public engagement activities and this might be a useful way of counteracting the negative portrayal of science in the mass media. Referring to the debate about genetically modified foods, Peter, a Reader in the Genetics theme, felt that PES could make a contribution through ‘dispelling improper views’. PES was a familiar concept to the health service researchers in the innovations theme, but they had more limited experience of engaging with the public in this way.

### A Mode 2 perspective on PPI and PES: ‘We weren't informing them, they were informing us’

Only one participant talked passionately about collaborating with the public and patients to share power and control over the process of knowledge production. She highlighted the need to build engagement in the research process and provide opportunities for service users (a term she preferred to ‘patients’) to be involved in shaping, critiquing and monitoring the research process. She explained that she considered engagement as involving agency, action and will on the part of the participant. She linked patient engagement to activism, whereby individuals trying to bring about change such as those campaigning for medication for people living with HIV/AIDS are proactive rather than passive and organized to gain influence through collective action. By contrast, she observed what she described as ‘rhetorical, tokenistic and box ticking’ approaches to PPI in the UK:

I don't think there is a lot of humility in the scientific community about their own need to be exposed, to…, because there is a certain elitism that floats around these circles in which people think they know the truth… So it's not that they're dying to get input from others and widen their perspectives. (Sue)

Sue and her colleague Jackie were also critical of PES as tokenistic and alluded to the deficit model of public understanding of science. Sue talked about her previous research experience outside UK health services research. Before coming to work as a health services researcher, she had worked in an international development context as both a researcher and health promoter alongside activists seeking to change policy and practice in sexual and reproductive rights, including HIV. She felt that the methods used in her previous research (mainly qualitative methods) were particularly well suited to participation.

Sue's perspective aligned with Wynne's description of a more democratic production of knowledge where the roles of researchers and the public are more fluid. While only Sue associated herself with a more participatory research tradition, other health service researchers shared her view of the elitist research culture within health care. Sam had observed a ‘natural resistance’ to the idea of involving patients. Jackie, another health services researcher agreed:

I think there is very much a normative ideal that actually professionals are the only ones that really have the authority and knowledge to write research protocols and undertake it. (Jackie)

Jackie felt that it should be possible to give the patient voice the same status as the professional voice in the research process. The commitment to sharing authority with other non‐academic stakeholders and working in partnership to produce knowledge expressed by Sue is aligned with Mode 2 forms of knowledge production. On the other hand, the need to retain control over the research highlighted by Jackie would fit more closely with Mode 1 research where power, funding and agenda setting remains within the Academy.

## Discussion and conclusions

The participants in this study made a clear distinction between PES (an activity that aims to communicate research findings to the public, engage the public with broader issues of science policy or promote a greater understanding of the role of science in society) and PPI (which they described as engagement with patient and carers in the conduct of research projects and programmes). Despite the PPI label, the public were not considered as part of PPI activity. PES was widely accepted but understood as the more traditional Public Understanding of Science; PPI was resisted by the biomedical scientists but accepted and sometimes embraced by health services researchers. However, even in the latter cases there was a resistance to the idea of power sharing. Mode 2 thinking in the production of scientific knowledge, fusing both PES and PPI, was exceptional and articulated by only one participant in this study; an individual with experience of activism and participatory approaches to research.

The study revealed considerable variability in activity and attitudes to PPI and PES amongst different types of researchers and between individuals. There was a clear divide between those who engaged in PPI and PES and who felt that they gained positive benefits from engagement activities, and those who were ‘going through the motions’ of engaging the public in their research, whether it be through PPI or PES activities. Most common was a pragmatic description of ‘scripted’ engagement with the public, patients and carers invited to ‘play the part’.

The health services researchers were more likely to be actively engaged in PPI activities, although the extent to which they thought this was valuable activity varied between individuals. The biomedical scientists had less experience of PPI although funding concerns had raised their awareness of the concept. Some individuals had been inspired by positive experiences of engaging with the public which led to a shift in their attitudes and practice. With the exception of two health services researchers, participants described a traditional model of public understanding of science motivated by the need to educate the public about science and improve accountability.

Previous researchers have found it helpful to use the concepts of Mode 1 and Mode 2 research to understand academic attitudes and practice.^11^, ^17^ While these studies have focused on a range of aspects of academic practice including agenda setting, interdisciplinary working, collaborations, outputs and dissemination, less attention has been paid to the relationship with the public and patients as key stakeholders in research. Similarly, the original texts on Mode 1 and Mode 2^3^, ^10^ make little mention of the public, patients and service users. Our analysis suggests that the different types of engagement activity undertaken with the public and patients align with different Modes. While participants were broadly accepting of the limited communication model associated with Public Understanding of Science (an activity more associated with Mode 1), they expressed greater resistance to types of engagement with the public and patients more associated with Mode 2. While PPI guidance is increasingly promoting Mode 2 knowledge production, the underlying attitudes of many researchers continue to reflect a Mode 1 set of values. For example, many interview participants were clear that only researchers can design and conduct research (particularly biomedical research) and that power and control by service users are ‘dirty words’. These findings support Ward *et al*.'s^14^ conclusions that researchers are not aiming for the higher rungs of Arnstein's ladder^18^ with regard to PPI. Our analysis suggests that it would be helpful to consider more active forms of engagement with the public and patients as part of the broader conceptualization of Mode 2 knowledge production.

The notion of a ‘blurring of the divide’ between science and society was only evident in one interview, where the researcher displayed a different set of values to those of the other participants in the study. As McKevitt^19^ observes, the production of knowledge is a moral and political process, in which the experiential knowledge of patients and the public can be seen to constitute a threat. Wilsdon *et al*.^20^ argue that the focus on the hardware of engagement (the how to, methods, approaches, guidelines etc.) rather than the ‘software’ of values, norms and codes that shape scientific practice helps to explain how the policy to promote PPI has made little impact on the attitudes of academic researchers. The authors highlight an aspirational vision captured in Alan Irwin's work^21^ on Citizen Science, in which scientists (what Wildson *et al*. term ‘citizen scientists’) start to see their own contribution to society as a key responsibility and as part of their working lives. As Wilsdon *et al*. prophesied in 2005:

We will end up with little more than the scientific equivalent of corporate social responsibility: A well‐meaning, professionalized and busy field, propelled along by its own conferences and reports, but never quite impinging on fundamental practices, assumptions and cultures.^20^

The data we report here provide further evidence of the maintenance of Mode 1 academic attitudes and values, despite the ‘changing currents on the surface’.^14^ In our study, while participant researchers were prepared to go through the motions in pursuing PPI and PES activities and listed the advantages of engagement, the interviews revealed underlying attitudes consistent with those reported in previous studies^14^ and an active resistance to sharing power and control in the process of knowledge generation. Instead, the ‘defensive power’ of the research community identified by Whitley^12^ and ‘pull back’ to Mode 1 from academic disciplines and institutions^11^ persist.

## References

[1] House of Lords Select Committee on Science and Technology . Science and Society, Third Report of Session 1999–2000, 2000, HL38. Available at: http://pubs1.tso.parliament.uk/pa/ld199900/ldselect/ldsctech/38/3801.htm, accessed 19 March 2014.

[2] Department of Health . Best Research for Best Health, 2006.

[3] Nowotny H , Scott P , Gibbons M . Re‐Thinking Science: Knowledge and the Public in an Age of Uncertainty. Cambridge: Polity Press, 2001.

[4] Wynne B . Public engagement as a means of restoring public trust in science – hitting the notes, but missing the music? Community Genetics, 2006; 9: 211–220. 16741352 10.1159/000092659

[5] Rowe G , Frewer LJ . Public participation methods: a framework for evaluation. Science, Technology and Human Values, 2000; 25: 3–29.

[6] Weldon S . Public engagement in genetics: a review of current practice in the UK, 2004 Available at: https://id503.securepod.com/nowgen/publication_media/nowgenreview3.pdf, accessed 19 March 2014.

[7] Nilsen ES , Myrhaug HT , Johansen M , Oliver S , Oxman AD . Methods of consumer involvement in developing healthcare policy and research, clinical practice guidelines and patient information material A Cochrane Review. The Cochrane Collaboration 2010, Issue 1. John‐Wiley and Sons. 10.1002/14651858.CD004563.pub2 PMC6464810 16856050

[8] Boote J , Telford R , Cooper C . Consumer involvement in health research: a review and research agenda. Health Policy, 2002; 61: 213–236. 12088893 10.1016/s0168-8510(01)00214-7

[9] Evans D . Patient and public involvement in research in the English NHS: A documentary analysis of the complex interplay of evidence and policy. Evidence and Policy, 2014; 10: 361–377.

[10] Gibbons M , Limoges C , Nowotny H , Schwartzman S , Scott P , Trow M . The New Production of Knowledge: The Dynamics of Science and Research in Contemporary Societies. London: Sage, 1994.

[11] Ferlie E , Wood M . Novel Mode of knowledge production? Producers and consumers in health services research. Journal of Health Services Research & Policy, 2003; 8: 51–57. 14596748 10.1258/135581903322405171

[12] Whitley R . The Intellectual and Social Organisation of the Sciences, 2nd edn Oxford: Oxford University Press, 2000.

[13] Thompson J , Ward P , Barber R *et al* Health researchers′ attitudes towards public involvement in health research. Health Expectations, 2009; 12: 209–220. 19392833 10.1111/j.1369-7625.2009.00532.x PMC5060486

[14] Ward P , Thompson J , Barber R *et al* Critical perspectives on consumer involvement in health research. Journal of Sociology, 2009; 46: 63–82.

[15] Stilgoe J , Lock S , Wilsdon J . Why should we promote public engagement with science? Public Understanding of Science, 2014; 23: 4–15. 24434705 10.1177/0963662513518154 PMC5753839

[16] Woolf SH . The meaning of translational research and why it matters. Journal of the American Medical Association, 2008; 299 : 211–213. 18182604 10.1001/jama.2007.26

[17] Pettigrew A . Distinguished Scholar Address to the Organisation and Management Theory Conference. Vancouver, BC: Division of the US Academy of Management, 1995.

[18] Arnstein S . A ladder of citizen participation. American Institute of Planners Journal, 1969; 35: 216–224.

[19] McKevitt C . Experience, knowledge and evidence: a comparison of research relations in health and anthropology. Evidence & Policy, 2013; 9: 113–130.

[20] Wilsdon J , Wynne B , Stilgoe J . The Public Value of Science: Or how to Ensure That Science Really Matters. London: Demos, 2005.

[21] Irwin A . Citizen Science: A Study of People, Expertise and Sustainable Development. London: Routledge, 1995.

